# Primary lipoma arborescens of the knee may involve the development of early osteoarthritis if prompt synovectomy is not performed

**DOI:** 10.1007/s10195-014-0295-x

**Published:** 2014-05-06

**Authors:** Luis Natera, Pablo E. Gelber, Juan I. Erquicia, Juan Carlos Monllau

**Affiliations:** 1 Hospital de la Santa Creu i Sant Pau, Universitat Autònoma de Barcelona, C/Sant Antoni Maria Claret 167, 08025 Barcelona, Spain; 2 Hospital Universitari Quirón Dexeus, Universitat Autònoma de Barcelona, C/Sabino Arana, 5-19, 08028 Barcelona, Spain

**Keywords:** Lipoma, Arborescens, Knee, Osteoarthritis, Synovectomy

## Abstract

**Background:**

Primary lipoma arborescens (LA) is a rare, benign intra-articular hyperplastic tumor that has been associated with osteoarthritis (OA). The aim of this study was to determine whether prompt synovectomy could avoid progressive joint degeneration in cases of primary LA of the knee.

**Materials and methods:**

A review of currently available literature about the disease was carried out. The clinical, histological and radiological records of a series of nine knees with primary LA diagnosed and treated between 2002 and 2012 were retrospectively reviewed. Eight of the knees had histological confirmation of LA and none had evidence of condropathy on the initial magnetic resonance image or degenerative changes at the initial radiographic examination.

**Results:**

At the final follow-up no evidence of OA was found in the three knees that underwent synovectomy when symptoms did not last more than 1 year. The five knees in which synovectomy was delayed developed progressive joint degeneration.

**Conclusion:**

In this series, primary LA of the knee involved the development of early osteoarthritis when prompt synovectomy was not performed. Timely synovectomy is strongly recommended, if not mandatory.

**Level of evidence:**

IV.

## Introduction

Primary lipoma arborescens (LA) is a rare, benign intra-articular hyperplastic tumor characterized by villous, polypoidal, and lipomatous proliferation of the synovium [1, 2]. Although some cases have been reported in the glenohumeral joint, the subdeltoid bursa, the elbow, the wrist, the hip and the ankle [3–6], nearly all the cases involve the suprapatellar pouch of the knee [7]. It is considered a primary benign tumor of unknown etiology [8]. Histology shows a hyperplastic process characterized by a diffuse replacement of the subsynovial tissue with mature fat cells [9]. This leads to prominent villous polypoidal proliferation of the synovium. Patients usually present with a chronic or recurrent painless swelling of the knee joint [2]. However, patients look for medical attention at different stages of the disease and in some cases, they may be referred by other specialists after unsuccessful conservative treatment and after having been misdiagnosed. This has a clear impact on their prognosis as the progression of the disease frequently leads to osteoarthritis (OA) over a short period of time. Although it is usually postulated that most cases of primary LA of the knee have similar presenting clinical characteristics [10], different clinical situations with different prognoses are presented in this series of nine cases (Table 1). The aim of this study was to determine if prompt synovectomy could avoid progressive joint degeneration in cases of primary LA of the knee.

**Table 1:** Details of seven patients with lipoma arborescens of the knee

Case	1	2	3	4	5	6	7
Age	39	53	53	37	34	57	40
Gender	Woman	Woman	Woman	Man	Man	Man	Woman
Additional conditions/situations	None	None	None	Marathon runner	-Lymphoma B	-High blood pressure	None
						-Dyslipidemia	
						-Obesity	
						-Smoking	
						-Alcoholism	
Trigger or particular debut	None	None	None	After a twist playing soccer	None	Sudden, oppressive and atraumatic left knee pain with a large effusion	None
Prior to surgery	12 months	37 months	30 months	4 months	11 months in the right knee	23 months	10 months
Prior to surgery Physical symptoms and Findings	Recurrent effusions and discomfort	Recurrent effusions, swelling and a large palpable supra-patellar mass	Recurrent effusions and anterior knee pain	Soft tissue palpable mass above the superior aspect of the patella and anterior knee pain	Recurrent effusions, pain and swelling in both knees	Oppressive left knee pain.	Pain, swelling and recurrent effusions
Laterality	Right	Right	Right	Left	Bilateral	Bilateral	Left
Treatment	AS	OS	2 AS. PFA*. 1 AS after PFA	AS	AS in both knees	OS in the left knee, no treatment in the right knee	AS
Results	Pain free. No effusions	Pain free. No effusions	Still pain. Still effusions	Pain free. No effusions	Asymptomatic in both knees	The symptoms of the left knee subsided almost completely. Still pain in the right knee	Pain free. No effusions
Surgical Findings	Bright yellow villi in the whole suprapatellar pouch	Large, pedicled and encapsulated tumor	Synovitic villi in the whole knee	Synovitic villi in the suprapatellar pouch	Synovitic villi at the suprapatellar pouch of both knees	An elongated diffuse synovial proliferation in the whole knee	Synovitic villi in the whole knee
Follow up	14 months	22 months	25 months	12 months	27 months in the right knee	29 months	44 months
Evidence of osteoarthritis	No	Yes	Yes	No	Yes, in the left knee	Yes, in both knees	No

*AS* arthroscopic synovectomy

*OS* open synovectomy

* *PFA* patellofemoral arthroplasty

## Materials and methods

**Fig. 1:**
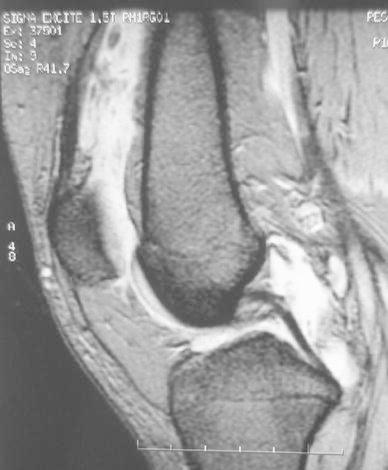
Sagittal reconstruction of the MRI showing villous-like projections invading the suprapatellar pouch with a fat signal intensity on T2

**Fig. 2:**
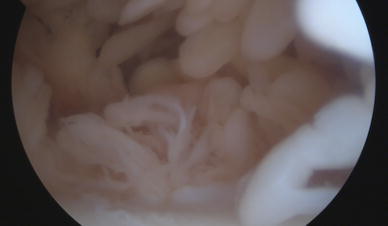
An arthroscopic view from the anterolateral portal of a villous synovitic mass in the suprapatellar pouch

**Fig. 3:**
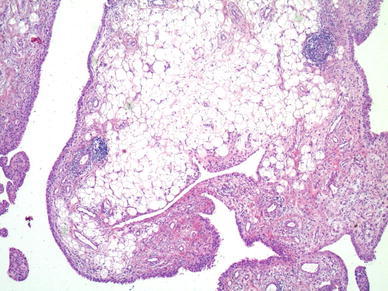
Histological view of a hyperplastic process characterized by a diffuse replacement of the subsynovial tissue with mature fat cells, causing villous expansion of the synovium. Inflammatory cells are observed around capillaries (haematoxylin and eosin ×30)

**Fig. 4:**
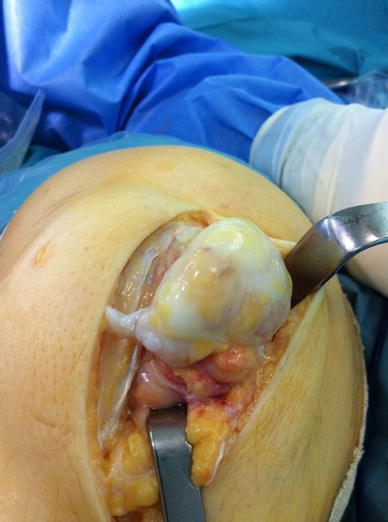
Large encapsulated tumor attached with a pedicle to the anterior aspect of the joint capsule at the level of the fat pad, seen during an anteromedial parapatellar arthrotomy

**Fig. 5:**
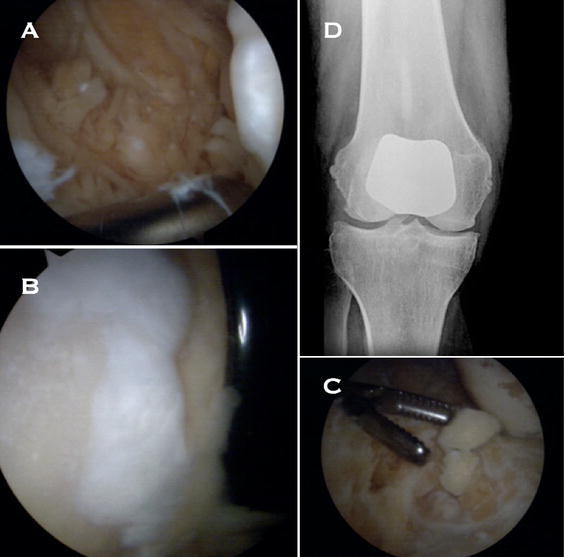
**a**, **b**, **c** Arthroscopic view of a right knee with a patellofemoral arthroplasty, subchondral bone exposure in the femoral condyle and diffuse synovitic proliferation. **d** AP view of patellofemoral arthroplasty

We retrospectively reviewed the clinical, histological and radiological records of patients diagnosed with primary LA of the knee and treated by two senior surgeons in three institutions between 2002 and 2012. We asked in the Pathology Departments of these three institutions for histological records coded as “lipoma arborescens”. We excluded cases involving other joints different to the knee, patients older than 60 years old, and cases where the initial magnetic resonance image (MRI) diagnosis of LA was accompanied by condropathy. The final population consisted of seven patients. Two of the patients had bilateral LA, so the total number of cases was nine. There were four men and three women with a mean age of 44.7 (34–57) years old. All of the patients had an MRI, which showed in all cases an exophytic mass in continuity with the synovium with villous-like projections invading the suprapatellar pouch with a fat signal intensity (Fig. 1) and no evidence of condropathy. Initial radiographs showed no evidence of degenerative changes in any of the cases. The establishment of the diagnosis of osteoarthritis was made by means of the observation in the X-ray of joint sclerosis, joint space narrowing and/or osteophytes. The diagnosis of secondary OA was established by means of the observation via X-rays of degenerative changes that were not present at the initial radiological examination. The main symptoms in all patients consisted of increasing and progressive discomfort, pain, swelling and recurrent effusions. The magnitude of the symptoms was not graded. We quantified the pain and the effusions in a dichotomic manner: present or absent. One of the patients had history of lymphoma-B and other of hyperuricemia. One of them said that the initial symptom was a sudden oppressive atraumatic knee pain with a large effusion, and another related the onset of symptoms due to a traumatic episode. The remaining patients did not relate the onset of symptoms to a particular situation. In eight of the nine knees, resection of the tumor was performed: six arthroscopic and two open. The two patients with bilateral LA underwent arthroscopic synovectomy: one of them in both knees and the other only in the left one, because, despite our recommendations, he rejected surgical treatment due to personal reasons. The timing of the synovectomy was agreed by the patient and the surgeon; and it was mostly related to the magnitude of the initial discomfort. The synovial tissue excised in the eight cases was afterwards sent for histological examination. The mean duration of the symptoms from debut until surgery was 19.13 months (4–37). The findings from the six knees that underwent arthroscopic surgery consisted of a marked villous proliferation of the synovial membrane and bright yellow and slightly firm villi on the whole suprapatellar pouch (Fig. 2). The histologic evaluation confirmed the diagnosis in all cases (Fig. 3). In one patient the excision could not be done arthroscopically because of the size, consistency and location of the tumor. A 60 × 45 mm mass was firmly attached to the anterior aspect of the joint capsule at the level of the fat pad, so a longitudinal anteromedial parapatellar arthrotomy had to be performed for a complete resection of the mass. The tumor was pedicled and encapsulated, as shown in Fig. 4. In another patient who underwent an arthroscopic synovectomy with no evidence of osteoarthritic changes, due to persistent and recurrent effusions, 15 months later another MRI revealed a remaining soft tissue mass in the suprapatellar pouch, but with concomitant patellofemoral degenerative changes at that moment. A second arthroscopic procedure was then performed. A pediculated synovial tumor with concomitant exposed subchondral bone in both the patella and femoral trochlea were found. A second synovectomy was performed. Eight months later the patient was still complaining of disabilities in daily life activities that was attributed to patellofemoral OA. A patellofemoral arthroplasty was then performed. Although the symptoms subsided for 6 months, recurrent effusions started again. A third arthroscopic surgery was subsequently performed. A diffuse synovitic proliferation with concomitant advanced degenerative changes in the medial tibiofemoral compartment were seen (Fig. 5). The mean follow-up from synovectomy until the last visit was 23 months (11–44). We arbitrarily established the threshold between prompt and delayed synovectomy at 1 year. We expose in detail the patterns of presentation and clinical conditions of this series of patients with primary LA (Table 1), in which we looked for a possible relationship between the time of synovectomy and the development of OA.

## Results

Synovectomy was performed in 88.89 % (8/9) of the knees. At the latest follow-up, 12.5 % (1/8) of the knees in which synovectomy was performed were pain and swelling free. Of the operated knees, 12.5 % (1/8) had recurrent effusions despite synovectomy. One of the patients with bilateral LA rejected synovectomy in the right knee, so it remained symptomatic. Of the knees that underwent synovectomy, 62.5 % (5/8) developed OA. One of the patients with bilateral LA, whose initial MRIs showed no evidence of condropathy in either knee, eventually developed OA in the knee that was not surgically treated. Of the operated knees, 37.5 % (3/8) underwent synovectomy in the first year after the onset of symptoms and 62.5 % (5/8) after the first year. All (3/3) of the knees that had undergone synovectomy when symptoms did not last more than 1 year showed no evidence of OA. All (5/5) of the knees in which synovectomy was delayed more than 1 year after clinical debut developed progressive joint degeneration.

## Discussion

This series of seven patients and nine knees diagnosed with primary LA with a variety of presentations of the same disease showed different prognosis conditions. The two most common complications were recurrence and development of secondary degenerative changes. The fact that the three cases where synovectomy was promptly performed did not develop degenerative changes, makes us conclude that timely excision of the tumor is strongly recommended, if not mandatory.

LA was first described in detail in 1957 [11]. It is an uncommon, benign intra-articular lesion of unknown etiology in which there is diffuse replacement of the subsynovial tissue by mature fat cells along with prominent villous transformation of the synovium. Although the knee is the joint most commonly involved, it has been described in other locations [3–6]. It most commonly affects people in the fourth and fifth decade of life [12]. Once considered more frequent in the male population, it is currently considered to be equally distributed in both genders [13]. The majority of cases are monoarticular [7]. In fact, there have been only a few cases of bilateral LA described in the literature [2, 11, 14–17], as were cases five and six of this series. A few reports have described polyarticular involvement [6, 17].

Almost all reports on primary LA of the knee have been single-case reports. Two short series previously published had six [18] and eight cases [19], respectively. A recent publication describes a group of 39 cases of secondary LA, as the consequence of chronic reactive changes in patients with OA [20]. In their series, only three of the cases had no evidence of OA. All of the patients in our series had an initial MRI with evidence of LA and no evidence of condropathy, as well as an initial radiographic examination without evidence of OA. We believe that primary and secondary LA should be considered as different entities.

Patients with LA usually have long-standing, slowly progressive swelling of the involved knee, which may be associated with effusion, decreased range of motion and pain. However, two patients in this series had started with a sudden onset of pain and effusion. A soft, painless, boggy swelling in the suprapatellar pouch can frequently be palpated. Due to the fact that the tumor is painless, patients usually seek medical evaluation after several years of mechanical symptoms, as was the case in some of the patients in this series. The laboratory tests are usually unremarkable and negative for HLA B27 and rheumatoid factor [8]. The joint aspirate is negative for crystals and cultures of the fluid are sterile [21].

Plain radiographs are generally normal during the first stages of the disease [16], but a soft-tissue density in the suprapatellar pouch can be observed if it is meticulously evaluated. In more advanced stages, subchondral bone erosions suggesting synovial invasion, cyst formation or secondary osteoarthritic changes can be seen.

The MRI is considered the gold standard for the diagnosis of LA [16]. It has a pathognomonic aspect consisting of an intra-articular synovial mass with frond-like architecture and a high signal intensity which is suppressed using fat-selective presaturation [16]. There is lack of enhancement after injection of gadolinium, which helps to exclude synovial inflammatory or neoplastic processes, and there are no magnetic susceptibility effects due to hemosiderin or calcification. MRI also allows for the correct evaluation of the size and extension of the tumor, accurate preoperative planning, evaluation of the state of the cartilage and effective follow-up while avoiding the need for synovial biopsy [22]. These findings in the MRI should be correlated with the histology that should be performed after resection, to confirm the diagnosis, and then medical professionals should be aware of recurrences that could have a clear influence on prognosis.

Macroscopically, LA has a frond-like appearance with numerous broad-based polypoid or thin papillary villi composed of fatty yellow tissue [7]. Histologically, the villi are composed of mature adipose tissue, and enlarged or congested hyperemic capillaries may be present [7]. The overlying synovial membrane may contain mononuclear chronic inflammatory cells and the synovial cells may seem to be enlarged and reactive with abundant eosinophilic cytoplasm.

An association between LA and OA has been postulated [14, 23], but the causal relationship between these two entities has not been fully clarified. It has been suggested that the long-standing synovial thickening effusions caused by repeated mechanical injury to the proliferated villi eventually lead to OA [2]. Subchondral cysts and bone erosions can also be observed in some patients [9], as were seen in case six of this report.

The severity of the degenerative changes might have some relationship with the duration of symptoms. Conversely, LA has been suggested to be secondary to OA in elderly patients [23]. Authors have classified this as a secondary type, which is much more common than primary cases. This secondary LA is thought to be a lipomatosis secondary to chronic irritation [24]. The same mechanism could be observed in cases of meniscal injuries, trauma and chronic synovitis. However, this is not an actual neoplasm, so it should not be considered as LA. Although the patient in case four related the onset of symptoms to a traumatic episode, due to the results of the MRI and histology compatible with LA, the traumatic injury was considered only a coincidence.

The recommended treatment for symptomatic LA is open or arthroscopic synovectomy [9, 20]. The election of one technique over the other mainly relies on the size of the tumor and on the personal experience and preferences of the surgeon. Those few previous reports that had performed the synovectomy arthroscopically reported favorable outcomes at 1 year [9] and 2 years [21, 25]. In this series, synovectomy was performed in 8 of the 9 knees. Recurrence of LA after surgical treatment is considered very rare [25]. In this scenario, the patient in case three, with several recurrences and who had undergone three arthroscopic synovectomies and a patellofemoral arthroplasty, is a very atypical case. In fact, despite the four surgical procedures, she is still symptomatic.

This retrospective case series of a low number of patients is, however, one of the largest series ever reported on primary LA. These patients were seen at different stages of the disease and treated with different surgical techniques. This heterogeneity might be a logical consequence of the different clinical expressions and the evolution of the disease observed among these patients.

We conclude that progressive joint degeneration could be prevented or at least delayed, if prompt synovectomy is performed.
